# Collaborative support for child abuse prevention: Perspectives of public health nurses and midwives regarding pregnant and postpartum women of concern

**DOI:** 10.1371/journal.pone.0281362

**Published:** 2023-03-06

**Authors:** Akemi Yokomizo, Hiroko Nagae, Rukmali Athurupana, Mikiya Nakatsuka

**Affiliations:** 1 Graduate School of Health Sciences, Okayama University, Okayama City, Okayama Prefecture, Japan; 2 Department of Nursing, Faculty of Health, Medical and Welfare Sciences, Kibi International University, Takahashi City, Okayama Prefecture, Japan; 3 Kameda University of Health Science, Kamogawa City, Chiba Prefecture, Japan; Indian Institute of Dalit Studies (IIDS), INDIA

## Abstract

Child abuse is a globally prevalent problem, and its numbers have continuously increased in Japan over the past 30 years. Prevention of child abuse depends on the support available to pregnant and postpartum women from the time of pregnancy. Public health nurses and midwives are expected to provide preventive support in cooperation, as they can support pregnant and postpartum women from close proximity and recognize their health problems and potential signs of child abuse. This study aimed to deduce the characteristics of pregnant and postpartum women of concern, as observed by public health nurses and midwives, from the perspective of child abuse prevention. The participants comprised ten public health nurses and ten midwives with five or more years of experience working at the Okayama Prefecture municipal health centers and obstetric medical institutions. Data were collected through a semi-structured interview survey and analyzed qualitatively and descriptively using an inductive approach. The characteristics of pregnant and postpartum women, as confirmed by public health nurses, included four main categories: having “difficulties in daily life;” “a sense of discomfort of not feeling like a normal pregnant woman;” “difficulty in child-rearing behavior;” and “multiple risk factors checked by objective indicators using an assessment tool.” The characteristics observed by midwives were grouped into four main categories: “mental and physical safety of the mother is in jeopardy;” have “difficulty in child-rearing behavior;” “difficulties in maintaining relationships with the surrounding people;” and “multiple risk factors recognized by an assessment tool.” Public health nurses evaluated pregnant and postpartum women’s daily life factors, while midwives evaluated the mothers’ health conditions, their feelings toward the fetus, and stable child-rearing skills. To prevent child abuse, they utilized their respective specialties to observe those pregnant and postpartum women of concern with multiple risk factors.

## Introduction

In Japan, the number of child abuse consultations handled by child guidance centers nationwide has continually increased in the past 30 years, exceeding 200,000 cases for the first time in the financial year (FY) 2020. Prevention of child abuse requires cooperation among multiple organizations [1], including medical, healthcare, and welfare institutions [2], and social welfare organizations [3]. In Europe and the U.S., Nierop et al. [4] discovered a relationship between stress during pregnancy and postpartum depression. They pointed out the importance of putting in specific efforts from the pregnancy period onward to ensure abuse prevention. In a randomized controlled trial, Olds et al. [5] found that prenatal home visits by nurses reduced child maltreatment and revealed the importance of maternal involvement during pregnancy. Furthermore, Ashraf et al. [6] state that health care providers can identify risk factors and signs of abuse in the medical setting. Additionally, referrals to community resources, parenting education, and other preventive measures must be incorporated into clinical practice. In Japan, there are several mother-child support programs for the prevention of child abuse, such as “Healthy Start Oita” [7] and “Suzaka Trial” [8], which provide seamless support during pregnancy, childbirth, and the postpartum period, per the characteristics of each region (for example, whether the government and obstetric hospitals can easily collaborate on a daily basis).

In 2011, the Okayama Prefecture started its operation of the “Contact system for support for mothers and children of concern during pregnancy” (hereinafter referred to as the “Okayama model”) [9], strengthening seamless support for pregnant and postpartum women of concern (PPWC) in collaboration with obstetric facilities and the community. The Okayama model is characterized by early recognition of risk factors at obstetric facilities by focusing on medical and social backgrounds and efforts to provide support in cooperation with multiple professions throughout pregnancy. According to the Ministry of Health, Labour, and Welfare’s FY 2018 Welfare Administration Report [10], the number of municipal consultation responses in the Okayama Prefecture was 850 in FY 2018 (3.54 consultations per thousand population per year), compared with 1,641 consultations in FY 2012 (6.29 consultations per thousand population per year), which represents a nationwide increase. However, the number of child abuse consultation responses has reduced. Kobayashi [11] analyzed child abuse and death cases and concluded that the key institutions for abuse prevention involved health and medical centers, hence stating the importance of cooperation between the two.

A multidisciplinary approach through partnership represents a standard for preventing child abuse [12, 13]. Early interventions with pregnant women at risk of child abuse are considered effective in preventing child abuse [14]. It is at administrative agencies, such as obstetric facilities where women are diagnosed with pregnancy and health centers where maternal and child health handbooks are issued, that public health nurses and midwives have contact with pregnant women at risk of child abuse or pregnant women about whom they feel a vague sense of alertness that “causes them to be concerned about something.” Public health nurses (PHNs) and midwives must share information to provide continuous support for preventing abuse if either institution identifies PPWC. Both PHNs and midwives provide ongoing support to expectant mothers: the PHNs through long-term life support for pregnant and postpartum women and their families, and the midwife in her role as a close supporter of women’s health. Hence, both are important partners in primary care for pregnant and postpartum women, and together they may be able to identify health problems and signs of potential child abuse.

Stolper et al. [15] stated that a feeling that “there is something wrong here,” a vague and intuitive sense of alertness, helps child health nurses become alert to situations that may lead to child abuse or maltreatment. Furthermore, a U.S. study by child abuse pediatricians (CAPs) reported that the diagnosis of child abuse is procured by a combination of intuitive responses elicited by family encounters and social information obtained outside those encounters [16]. Due to differences in their respective specialist disciplines, PHNs and midwives may have discrepancies regarding the identification of PPWC. Clarifying these differences is helpful for PHNs and midwives to understand each other’s perspectives and cooperate.

There are individual differences in the ability of health visitors to find and contact medically and socially high-risk pregnant women, depending on the person in charge [17]. There are cases wherein the affected individual may not receive support. Adachi et al. [18] clarified that whether a pregnant woman needs support is determined by the competence of the PHN. Similarly, it has been reported that there are individual differences in the ability of obstetric nurses to assess the risk of abuse depending on whether or not they have experience in caring for mothers about whom there are concerns related to child maltreatment [19]. Differences in the quality of their support (such as being able to recognize the risk of abuse) are influenced by their years of experience [20]. Experienced PHNs and midwives may have some standard assessment points when determining whether they are “concerned” about pregnant women and need to provide support. Clarifying these points will reduce the likelihood of such cases being overlooked due to individual abilities.

The latest research on child abuse prevention has focused on the characteristics of at-risk pregnant and postpartum women and the social and medical factors related to child abuse. So far, no research has elucidated the intuitive concern to pay attention to situations that may lead to child abuse from the perspective of PHNs and midwives who provide direct support to pregnant and postpartum women.

Therefore, this study aimed to clarify the characteristics of PPWC, as observed by experienced PHNs and midwives, in support of child abuse prevention. Our findings will promote abuse prevention and support from early pregnancy, resulting in the PHNs and midwives working together to ensure that these women in need of support are not overlooked.

### Operational definitions

#### Pregnant and postpartum women of concern

The implication of PPWCs is that the PHNs and midwives feel concerned about them while providing support during pregnancy and worry about the possibility of issues leading to child abuse.

#### The perspective of child abuse prevention

This refers to the perspectives of PHNs and midwives regarding awareness of the risk of child abuse due to the background factors, attitudes, and moods of pregnant and postpartum women, and on starting preventive support early on.

## Materials and methods

### Research design

This was a qualitative descriptive study with an inductive approach.

### Research participants

The participants included PHNs and midwives working at municipal health centers and obstetric medical institutions actively utilizing the contact system for the Okayama model in operation in the Okayama Prefecture. We requested cooperation in the study by written and verbal means from the general PHNs and the director of the nursing department of each institution. We also received recommendations of those who have had five years or more of experience in supporting mothers and children, including child abuse prevention.

### Data collection

The participants’ data were collected from August to November 2019. Semi-structured interviews were conducted based on an interview guide with the PHNs and midwives who agreed to participate at locations that were designated by the participants. Face-to-face interviews were conducted by the first author. The interview guide was developed after discussion with the faculty and graduate students in the field of adult and child health nursing, specializing in maternal support. During the interviews, the interviewees were asked to recall cases and situations where they felt “concerned” about pregnant and nursing mothers under the premise of abuse prevention and to describe their experiences and reasons for feeling concerned. The duration of the interviews with the PHNs was 54 minutes 46 seconds ± 10 minutes 59 seconds (mean ± SD) and with the midwives was 55 minutes and 16 seconds ± 10 minutes and 15 seconds (mean ± SD). All interviews were recorded on an integrated chip (IC) recorder with the consent of the participants and were transcribed verbatim, maintaining anonymity.

### Data analysis

Data were analyzed using qualitative inductive analysis methods. During the analysis, personally identifiable information was anonymized. Data analysis was conducted simultaneously with data collection, with interviews transcribed immediately after each interview. The interview transcripts were cross-checked among the researchers. The contexts of the concerns that the PHNs and midwives felt about expectant mothers were extracted from the transcripts. Coding was performed while considering these contexts so that the meaning of the narratives could be understood, and subsequent categorization was carried out based on the similarities and differences. After aggregating similar categories and examining their relationships, the categories were grouped. The codes were extracted then compared by two co-researchers with experience in qualitative research. The authors specialize in community nursing, and their expertise in supporting children and their families to live safely and healthily in the community was helpful in capturing concerns about mothers during the coding process. When opinions differed during the categorization process, the researchers repeatedly reviewed the results until a consensus was reached. They checked for any gaps between the intentions of the participants and the interpretation of the data, and presented the results to the participants to confirm the accuracy of the content.

### Ethical considerations

This research was approved by the Institutional Review Board of the Okayama University Graduate School of Health Sciences (D19-1). We briefed the participants verbally and in writing regarding the study’s purpose, the provision of voluntary and free withdrawal from participation, protection of personal information ensuring anonymity of the data provided, data storage method, and information regarding the publication of research results. After this, written consent was obtained.

## Results

### Overview of research participants

The research participants were ten PHNs working at municipal health centers in the Okayama Prefecture and ten midwives working at obstetric medical institutions, comprising 20 people in total. The number of years of experience as PHNs was 21.0±5.4 (mean±S.D.) years and 22.0±11.3 (mean±S.D.) years as midwives (Table 1).

**Table 1:** Background of the participants.

	Public Health Nurses (n = 10)	Midwives (n = 10)
**Age**		
Under 35 years	1	2
35–39 years	0	2
40–49 years	7	1
50 years and older	2	5
**Number of years of experience**	21.0±5.4[7–28]^a^	22.0±11.3[6–40]^a^
**Annual number of births at affiliated worksite**		
Less than 100	1	
100 to 199	3	
200 to 299	2	
300 and over	4	
**Number of deliveries at affiliated worksite **		
Less than 100		1
100 to 499		5
500 to 999		1
1,000 and over		3

This table demonstrates the age of the PHNs and midwives, their years of work experience, the annual births reported by PHNs, and the deliveries reported by the midwives at their respective affiliated worksites.

^a^Mean±S.D. [range]

### PPWC as seen by PHNs

We extracted four main categories, 12 subcategories, 32 subordinate categories, and 168 codes during the data analysis (Table 2).

**Table 2:** Characteristics of pregnant and postpartum women of concern as observed by public health nurses.

Main Category	Subcategory	Subordinate Category	ID
**Difficulties in daily life circumstances**	Daily life foundations are unstable	Having economic instability	A,B,C,E,H
		Difficulties in getting accustomed to the area due to transfer	D,E,G
	Lack of ability to support parents’ families	Parents’ households are in financial distress	E,H
		Family members at parents’ households have health issues	H,G
	Difficult to receive support from surrounding people	Poor relationship with parents	B,C,E,H
		Refusing support or involvement	B,C,D,I
		Having nobody to rely upon except parents	E,G
	Cannot envision life plan after giving birth	No planning for pregnancy	A,B,C,E,F,G,I,J
		Cannot envision daily life after giving birth	B,I,H
		No progress in preparations for life after giving birth	H,I
	In a dirty living environment	Garbage scattered in room and not cleaned up	G,H
		Unsanitary child-rearing space	A,H
**PHNs’ discomfort with the mannerisms of the pregnant women**	Difficult to communicate with	No progress in verbal exchange, and feeling that no conversation is established	A,B,C
		Poor facial expression	B,E,J
		Few remarks initiated by the individual	A,B,C,D,H,J
		Insufficient comprehension	C,E,J
	Having a unique way of thinking and mood	Having particular views on pregnancy and childbirth style	D,H
		Dressing in an unkempt manner	G
		Having particular views on unique health methods and ways of thinking	F,H
		Having unique mood	A,C,F,G,H
	Having mental instability	Having risk of mental instability	A,B,E,F,H,J
		Having emotional instability	A,D,F,H
**Have difficulty in child-rearing behavior**	Feelings are not directed toward the fetus/child	Having behaviors and attitudes that do not celebrate pregnancy	A,B,E,F,G,H,I
		Being unable to feel affection for fetus/child	B,E,F,G,I
		Not showing care and attention toward child	B,I
		Not changing drinking or smoking habits for fetus	B,G,I
	Having inappropriate child-rearing attitude toward older child	Having cold attitudes toward the older child	F,H
		Being unable to take care of older child	G,H,I,J
	Feeling unsure about child-rearing techniques	Feeling anxious about child-rearing and not feeling confident	B,F,G
		Having insufficient child-rearing skills and knowledge	B,C,G,H
**Have multiple risk factors recognized by an assessment tool**	Multiple risk factors checked by an assessment tool	Multiple risk factors checked by organization’s own risk indicators	A,B,C,J
		Multiple risk factors checked through a common form: “Contact form for support for mothers and children of concern during pregnancy”	A,B

This table demonstrates the four categories identified from the interviews with PHNs: have “difficulties in daily life;” “a sense of discomfort of not feeling like a normal pregnant woman;” “difficulty in child-rearing behavior;” and “multiple risk factors checked by objective indicators using an assessment tool.”

#### Difficulties in daily life circumstances

This category covers the health workers’ awareness that the pregnant or nursing mother had difficulties in her family background, living environment, socioeconoomic background, history, etc., and that stable living conditions were not in place, which caused them to be concerned about the mother’s situation. This category comprised five subcategories: “daily life foundations are unstable,” “lack of ability to support parents’ families,” “difficult to receive support from surrounding people,” “cannot envision life plan after giving birth,” and “in a dirty living environment.”

The findings showed that the pregnant and postpartum women’s unstable financial conditions and “difficulties in getting accustomed to the area due to transfer” resulted in unstable daily life foundations. Furthermore, even if they wanted their parents’ support, their situations showed a lack of ability to support the family home through factors such as the “lack of ability to support parents’ families” due to “financial distress” and “health issues” among the family members at the parents’ households. Furthermore, there were cases where the pregnant or parturient women refused “support or involvement” from PHNs or surrounding people. These women refused counseling when they were informed that they were pregnant and tried to return home after receiving the Mother and Child Health Handbook. They also had a “poor relationship with their parents” due to histories of childhood abuse and “having nobody to rely upon except parents”, which resulted in it being “difficult to receive support from surrounding people.” There were also cases where women were pregnant or parturient without plans for the daily life arrangements necessary for pregnancy or childbirth. Thus, a concern was raised regarding those who could not “envision a life plan after giving birth.” Furthermore, there were concerns raised by PHNs regarding “difficulties in daily life” among these women or their socioeconomic backgrounds as they lived in “dirty living environments”, with “garbage scattered in their rooms” without being cleaned or “unsanitary child-rearing spaces.”

#### PHNs’ discomfort with the mannerisms of the pregnant women

This category represented the discomfort that PHNs felt toward pregnant and postpartum women during their interactions at the time of pregnancy notification due to the unique mood or behavior of the women presented. This involved three subcategories: “difficult to communicate with,” “having a unique way of thinking and mood,” and “having mental instability.”

Concerns about “having a unique mood” were mentioned, with PHNs stating, “I am not exactly sure, but intuitively feel concerned” (PHN:C) about the unusual appearance of the woman’s hair or their complicated relationships with their companions at the time of pregnancy notification. There were also concerns about “having particular views on pregnancy and childbirth style,” such as a strong desire for a painless home delivery and “having particular views on unique health methods and ways of thinking.” These involved the beliefs of women wanting to control chronic illnesses through alternative healing powers. PHNs also mentioned concerns regarding the women “having mental instability,” such as “emotional instability,” as they had sad facial expressions, cried easily, or were easily swayed by their symptoms of mental illness. Meanwhile, PHNs witnessed cases wherein the pregnant women did not speak a word during the interview at the time of pregnancy notification, and their parents answered all questions. Sometimes, the conversations lacked factual statements regarding financial aspects, childcare supporters, and chronic illnesses, leading to “few remarks initiated by the individual.” These factors indicated “discomfort of not feeling like a normal pregnant woman,” with the PHNs sensing that it was “difficult to communicate with” the women due to their “poor facial expressions” or lack of “progress in verbal exchange” stemming from their inconsistent words and actions.

#### Have difficulty in child-rearing behavior

This category indicated concerns by PHNs that pregnant and postpartum women may not be able to perform appropriate child-rearing behaviors due to their lack of interest and involvement in their fetuses or babies, and their lack of confidence and skills in raising them. This was composed of three subcategories, namely, “feelings are not directed toward the fetus/child,” “having inappropriate child-rearing attitude toward the older child,” and “feeling unsure about child-rearing techniques.”

Concerns raised by PHNs included the women “having behaviors and attitudes that do not celebrate pregnancy,” with the nurses mentioning that the women did not view the pregnancy positively because it was unwanted. One PHN mentioned that the women “were unable to do the other things they wanted to do because of the pregnancy” (PHN:G). Other aspects included not finding their child cute and “being unable to feel affection for fetus/child,” resulting in the women strongly prioritizing themselves and “not changing drinking or smoking habits for the fetus.” Moreover, pregnant women expressed feelings of not being interested in or concerned about the fetus/child, such as not hugging the child even when it cried and “not showing care and attention toward the child.” PHNs also raised concerns about the women “having cold attitudes toward the older child” by ignoring their other biological children or step-children and treating them in an aggressive manner. This was described in the following narratives: treating the older child as if they are dirty and not letting him/her touch the baby, intentionally ignoring him/her even when he/she cried, and brushing away the step-children when they came near the baby. In addition, the PHNs expressed concern about the women “being unable to take care of the older child,” for example, through cleanliness and health management, due to insufficient child support. These women were viewed as “having inappropriate child-rearing attitude toward the older child.” A typical example was:

The woman would say that they are pregnant and they are having a difficult time, so they cannot do household chores, and they would ask the older sister in the upper grade of elementary school to even skip school to do household tasks or take care of the baby (PHN:J).

Meanwhile, PHNs mentioned concerns about the women “feeling anxious about child-rearing and not feeling confident.” One participant mentioned that although the pregnant or parturient woman felt affectionate toward the fetus or child, they are “ultimately not giving affection, or because they do not receive affection themselves, they do not know how to do it and feel anxiety” (PHN:B). There was also concerns about the women “feeling unsure about child-rearing techniques” because they did not know how to raise their children as they had “insufficient child-rearing skills and knowledge.”

#### Have multiple risk factors recognized by an assessment tool

This category indicated the PHNs’ concern toward those pregnant or parturient women with multiple risk factors for child abuse from objective indicators, such as medical records used for interviews or checklists within the organization. This category was composed of “multiple risk factors checked by an assessment tool.”

There are concerns of the woman being at high risk when there are multiple factors on the contact form for support for mothers and children [of concern during pregnancy] from the obstetrics facility, such as a history of mental illness, being in a step-family, or being of advanced maternal age. (PHN:A)

### PPWC as seen by midwives

We extracted four main categories, nine subcategories, 33 subordinate categories, and 178 codes during the data analysis (Table 3).

**Table 3:** Characteristics of pregnant and postpartum women of concern as seen by midwives.

Main Category	Subcategory	Subordinate Category	ID
**Mental and physical safety of mother is in jeopardy**	Having risk of childbirth that jeopardizes maternal safety	Being careless about managing own physical condition	a,b,c,f,i,j
		Suspected domestic violence leading to miscarriage	a,d,f,g
		Being fixated on desired childbirth style	b,d,i
		First visit or hospital transfer after 30 weeks of gestation	h,j
		Previous experience of childbirth with no prenatal care	a
	Having mental instability	Feeling depressed and having sad facial expressions	b,c,j,h
		Having emotional instability	d,h,f,g
		Having history of mental illness	a,c,d,g
**Have difficulty in child-rearing behavior**	Feelings are not directed toward the fetus/child	Not showing care and attention toward child	a,b,c,g
		Trying to prioritize self over raising child	a,b,d
		Being unable to feel affection for child	a,c,j
		Being unable to accept pregnancy	b,h
	No progress in preparations for childbirth and life after giving birth	Using or considering livelihood protection or hospitalized midwifery system due to financial instability	a,c,d,e,f,j
		Not feeling like she will become a mother	e,h,j
		Not having a fixed residence	a,f
		No progress in preparation of items for childbirth	e,h
	Having inappropriate child-rearing attitude toward older child	Having cold attitudes toward the older child	b,i,g,j
		Older child is unkempt in appearance	c,e
	Difficulties due to child-rearing not progressing as expected	Feeling confused due to being unable to raise child as expected	a,b,d,i,j,h
		Lack of knowledge regarding child-rearing is evident	b,d,f,i,j
		Having many minor questions	b,d,h
		Fixating on child-rearing by the book	b,d,h,j
		Feeling unsure about child-rearing techniques	j,h,g,b
**Have difficulties in maintaining relationships with surrounding people**	Difficult to receive cooperation from surrounding people	Having minimal cooperation from husband or partner	c,g,j,I,d
		Being unkempt and having a detached mood from surrounding people	e,f,j,d
		Refusing to have other people step in to individual affairs	a,e
		Having a small number of visitors and visits during hospitalization	a,j,e
		Having unreliable parents due to disagreement or history of abuse	h,c,j
	Difficult to communicate	Few remarks initiated by the individual	d,j,e,b
		Having disjointed conversations	b,d,f,e
		Poor comprehension	a,e
**Have multiple risk factors recognized by an assessment tool**	Multiple risk factors checked by an assessment tool	Multiple risk factors checked through a common form: "Contact system for support for mothers and children of concern during pregnancy"	a,b,c,d
		Multiple risk factors checked by organization’s own risk indicators	b,e,h,j

This table demonstrates the four categories identified from the interviews with midwives: “mental and physical safety of mother is in jeopardy;” “have difficulty in child-rearing behavior;” “have difficulties in maintaining relationships with surrounding people;” and “have multiple risk factors recognized by an assessment tool.”

#### Mental and physical safety of mother is in jeopardy

This category referred to midwives feeling that pregnant and postpartum women were at risk of being unable to have safe births. It comprised two subcategories, namely “having risk of childbirth that jeopardizes maternal safety” and “having mental instability.” Midwives mentioned concerns regarding cases where they were unable to keep track of the pregnancy until just before giving birth, such as “previous experience of childbirth with no prenatal care,” “first visit or hospital transfer after 30 weeks of gestation,” or when the pregnant or parturient woman was “being fixated on a desired childbirth style,” such as a painless home delivery, without considering her body’s safety. Furthermore, midwives felt concerned about the mother’s mental and physical safety being in jeopardy if they had negative emotional expressions, like “feeling depressed and having sad facial expressions,” or psychological issues, like “having mental instability.”

#### Have difficulty in child-rearing behavior

This category referred to the midwives’ concerns about pregnant or parturient women experiencing difficulties in raising children because of their lack of affection or involvement with their children and siblings. It comprised four subcategories: “feelings are not directed toward the fetus/child,” “no progress in preparations for childbirth and life after giving birth,” “having an inappropriate child-rearing attitude toward older child,” and “difficulties due to child-rearing not progressing as expected.”

Midwives felt concerned that the women did not have affection toward their children as they were “unable to accept the pregnancy” or that they were not thinking about their daily life after childbirth at all and making “no progress in preparations for childbirth and life after giving birth” because they did not want to acknowledge that they would become mothers.

Furthermore, midwives mentioned concerns about difficulties occurring when child-rearing was not progressing as expected, such as “feeling confused due to being unable to raise the child as expected.” This was because the challenges of raising a child exceeded the women’s expectations and were different from what they had anticipated, as seen in the statement: “She became pregnant with infertility treatment, and it was all well and good at childbirth, but she cried about not thinking it would be so tough, and she does not want to take care of the baby” (MW:d). The women were also “fixating on child-rearing by the book” and “having many minor questions,” as seen in the statement: “They would thoroughly read through the child-rearing book and immediately contact nurses when something does not go exactly as mentioned and what they should do” (MW:d). Midwives also expressed concern about “difficulty in child-rearing behavior.” For instance, they witnessed pregnant or postpartum women using harsh words or actions toward their older child in the waiting rooms or wards, with the women “having cold attitudes toward the older child,” or “having inappropriate child-rearing attitudes toward older child” when the “older child is unkempt in appearance.”

#### Have difficulties in maintaining relationships with surrounding people

This category referred to midwives’ concerns that the women lacked an immediate supporter who could cooperatively help them, rejected support, or did not have any relationship with surrounding people. This comprised two subcategories: “difficult to receive cooperation from surrounding people” and “difficult to communicate.” Midwives felt concerned about the women engaging in child-rearing and household work by themselves after being discharged from the hospital due to “having minimal cooperation from husband or partner,” “unreliable parents due to disagreements or history of abuse,” or “having a small number of visitors and visits during hospitalization.” One participant mentioned, “The husband has night shifts, so the woman is always thinking about how to stop [the baby from] crying” (MW:g). Furthermore, the midwives expressed concern about the pregnant or parturient woman finding it “difficult to receive cooperation from surrounding people” because they “refusing to have other people step in to individual affairsrefuse to let others into their personal matters:” “They have an atmosphere of not wanting others to get involved, such as, “It is fine, I will do everything by myself’” (MW:a).

The midwives also expressed concern about “difficulties in maintaining relationships with surrounding people” because of difficulties in communication, such as in cases where there were “few remarks initiated by the individual,” as seen in the statement: “The woman usually does not have conversations with the midwife, and even if she comes [to the medical examination] with her mother, it is just the mother talking, and there are few reactions from the woman herself” (MW:j). There were also instances of “having disjointed conversations,” as seen in the statement, “The answer I get is slightly different from what I asked. She likes giving lots of answers to things she is interested in. She does not respond for the important parts” (MW:d).

#### Have multiple risk factors recognized by an assessment tool

This category referred to concerns regarding pregnant and postpartum women with multiple risk factors related to child abuse, using objective indicators such as maternity interviews during initial visits and checklists used within facilities during maternity examinations. This category comprised “multiple risk factors checked by an assessment tool.” A typical example is as follows:

I try to make a template and pick up people who are likely to require follow-ups during pregnancy so that I can continuously do so […] I try to keep an eye on people throughout their pregnancies when they have several risk factors (MW:h).

## Discussion

### PPWC as seen by PHNs and midwives

Characteristics of how PHNs and midwives viewed PPWC included determining the target women by maximizing their specialist strengths in their respective professions and viewing PPWC as targets for support.

Common aspects of PPWC, as seen by both PHNs and midwives, were those considered so-called specified pregnant women [21], who did not display affection toward the fetus/child because of undesired or unexpected pregnancies; those lacking “support from surrounding people” as they were unmarried, single mothers, or not obtaining cooperation from the husband; and those with child-rearing problems, such as “having inappropriate child-rearing attitudes toward the older child.” Obstetrics and medical institutions are expected to provide information on specified pregnant women to administrative institutions as support targets for abuse prevention. This study showed that both PHNs and midwives commonly perceived specified pregnant women as targets for support and were conscious of their relationship with pregnant and postpartum women.

Furthermore, PHNs were characterized by their focus on the daily life background, child-rearing ability, and environment of the pregnant and postpartum women with high social risk, such as “having a family background where parents’ home cannot be relied upon” or having difficulties in daily life due to unstable daily life foundations. The nurses viewed childcare in the context of a stable lifestyle. Previous research [22, 23] reported that child-rearing supporters for pregnant and postpartum women from pregnancy to the first month after childbirth were mainly immediate family members, such as the women’s husband, mother, and mother-in-law. The present results indicate that the absence of people to rely on can pose a threat to the mental and physical stability of pregnant women.

Meanwhile, midwives focused on maternal health management and stable fetal and child-rearing skills for safe delivery. Their concerns were regarding pregnant and postpartum women with medical risks, such as their mental and physical safety, and those who “have difficulty in child-rearing behavior” that may continue after being discharged from the hospital. This included those not thinking at all about life after childbirth and making “no progress in preparations for childbirth and life after giving birth,” those with anxiety due to mental and physical changes from lifestyle changes around the child experienced in the early postpartum period, and those with a lack of knowledge regarding breastfeeding skills and childcare. Thus, the midwives’ perspectives regarding child-rearing behaviors focused on life after being discharged from the hospital. In this manner, the strengths of the specialties [24] of PHNs, who are close to the community, and midwives, who specialize in pregnancy and childbirth, were maximized when identifying the pregnant and postpartum women.

Specified pregnant and postpartum women with high social risks have many overlapping elements, and they may also include pregnant women with high medical risks [25]. Thus, child abuse may be prevented by supporting pregnant women who may or may not require child-rearing support due to various factors, whether or not these factors may lead to child abuse. Sharing feelings of “concerns” toward pregnant and postpartum women based on the perspectives of PHNs and midwives may allow pregnant women to be continuously given support without being overlooked. The characteristics of a PHN, who captures the daily life background and child-rearing environment of PPWC, as well as those of a midwife, who focuses on maternal safety and child-rearing skills, need to be mutually understood by the other profession. These two professionals also need to notice the risks that lead to abuse, sharing this information among supporters as soon as they become aware.

Matsubara [26] indicated that the mothers and children deemed “of concern” by the PHNs in 18-month child health examinations might be concerned about unique aspects that do not fit into the general image held by PHNs, or aspects concerning a minority of people. Even among the PPWC, as seen by the PHNs in this study, the PHNs felt that certain women with unique moods or thoughts “have the discomfort of not feeling like a normal pregnant woman.” Ozawa et al. [27] indicated that the job of a PHN is to guide the health conditions of an individual toward a better direction and to foster their ability to maintain an active life. When a PHN feels “concerned for some reason,” then this indicates that the possibility of a problem is present and that the individual needs assistance. Furthermore, valuing the “concerning aspect” and process of verification will lead to an improvement in the quality of on-site practice. Therefore, future studies should verify whether a pregnant or parturient woman who is considered “of concern” in this study truly requires support, as well as determine the effect of providing support from the point where a concern for the woman arises.

### Suggestions for collaborative support to PPWC provided by PHNs and midwives

Methods for determining PPWC for both the PHNs and midwives included identifying “concerning” aspects not only from subjective information, such as behavior and attitude during interviews with the women, but also from objective information, such as the questionnaire used at the time of the interview and contents of the risk assessment index. Since 2011, Okayama Prefecture has been using the “Contact form for support for mothers and children of concern during pregnancy” as a communication tool between obstetric medical institutions and administrative institutions using the Okayama model [9]. This system has been in operation throughout the Okayama Prefecture, with the current study also including using a contact form that was unique to the Prefecture. In recent years, risk assessments have been conducted using indicators that were independently created by child abuse prevention committees within obstetric and medical institutions. The importance of continuous collaborative support at related institutions, such as medical and administrative institutions, has been recognized [28]. Wada [29] stated that in the field of obstetrics medical care for pregnant women, there are individual differences in the “concerns” of midwives depending on the staff in charge. However, preparing a standard framework and dealing with issues as a team instead of relying on individual sensibilities can change the response from “being concerned” to “noticing” issues. Furthermore, activity reports regarding initiatives for preventing child abuse in the perinatal medical fields indicated the effectiveness of systematic risk determination using checklists and the promotion of collaboration with health institutions [30]. Both PHNs and midwives using common risk assessment indicators allow for the possibility of noticing that PPWC may require support. Consequently, if both the PHNs and midwives share their observations, they will not overlook the PPWC in need of support, establishing a support system from an early stage and serving as the first step for providing continuous monitoring support [13].

Yamaguchi et al. [31] reported the following as examples of midwives’ concerns when a mother and child in the early postpartum period are discharged from the hospital: “single concern,” such as support from surrounding people, child-rearing techniques, and mental illness complications; and “multiple concerns,” which are combinations of such single concerns. “Single concerns” represent issues that mothers and children generally encounter, and care tends to focus on these concerns such that it is easy to provide care that is tailored to each mother and child. Meanwhile, “multiple concerns” are highly subjective and involve various factors, including the environment. Thus, there is a wide variety of care recipients and content, and time is required until the effects begin to improve. The present results reveal that both PHNs and midwives viewed those who “have multiple risk factors recognized by an assessment tool” as PPWC and demonstrated their respective expertise in responding to such women with multiple factors, suggesting the importance of collaborative support. A wide range of factors, such as inadequate prenatal check-ups [32], poverty, and housing instability [33], were consistent with previous studies. These findings suggest the importance of both parties demonstrating their expertise and working together to support pregnant and nursing mothers who are concerned about a combination of these factors. Yamazaki [34] indicated a need for related professions to gain a common understanding regarding contact methods between medical and health institutions and that information sharing will progress effectively through a mutual understanding of the differences in perceptions between the PHNs and midwives.

Meanwhile, regarding collaboration issues between the PHNs and midwives, Hattori et al. [35] reported that the midwives were worried about whether their perspectives on “mothers and children of concern” were appropriately communicated to the PHNs. Many reports mention that turf issues and the division of roles hinder good inter-agency collaboration [36, 37]. Furthermore, Karata et al. [38] stated that feedback of information from other institutions is essential for developing collaborations after the nurses at obstetrics and medical facilities provide information on “parents and children of concern.” In the future, it will be necessary to determine how the PHNs and midwives view each other’s characteristics regarding PPWC and how they provide support while investigating the ideal way for effective collaboration between the two professions.

### Research limitations and future issues

This study has some limitations. Only ten PHNs and ten midwives participated, and their workplaces were limited to a single prefecture. Furthermore, we targeted PHNs and midwives with five or more years of experience in this study. However, both groups had an average of over 20 years of experience. Hence, their identification of PPWCs likely differed according to their years of experience. Future tasks involve the development of indicators to support the prevention of child abuse from the early stages of pregnancy without overlooking pregnant and postpartum women needing support, regardless of the number of years of experience of the nurses and midwives. Moreover, the construction of an effective collaborative support model for PHNs and midwives is needed to prevent child abuse.

## Conclusion

The study’s results revealed that each professional had their perspectives of determining target women by using their respective specialties and that had a common perspective of determining specified pregnant women as PPWC. PHNs focused on childcare in a stable lifestyle, wherein the foundations involved the daily life backgrounds of the pregnant or parturient woman. Contrarily, midwives focused on the health management of mothers and stable fetal and child-rearing skills, alongside perspectives on child-rearing behavior after discharge from the hospital. Future research must determine how each professional views the other’s characteristic perspectives for providing support, alongside investigating the ideal way for effective collaboration between these two professions.
